# A Critical Comparison between Flow-through and Lateral Flow Immunoassay Formats for Visual and Smartphone-Based Multiplex Allergen Detection

**DOI:** 10.3390/bios9040143

**Published:** 2019-12-12

**Authors:** Georgina M. S. Ross, Gert IJ. Salentijn, Michel W. F. Nielen

**Affiliations:** 1 Wageningen Food Safety Research, Wageningen University & Research, P.O. Box 230, 6700 AE Wageningen, The Netherlands; gert.salentijn@wur.nl (G.I.S.); michel.nielen@wur.nl (M.W.F.N.); 2 Laboratory of Organic Chemistry, Wageningen University, Helix Building 124, Stippeneng 4, 6708 WE Wageningen, The Netherlands

**Keywords:** flow-through immunoassay, lateral flow immunoassay, food allergen, multiplex, smartphone analysis, carbon nanoparticle labeling

## Abstract

(1) Background: The lack of globally standardized allergen labeling legislation necessitates consumer-focused multiplexed testing devices. These should be easy to operate, fast, sensitive and robust. (2) Methods: Herein, we describe the development of three different formats for multiplexed food allergen detection, namely active and passive flow-through assays, and lateral flow immunoassays with different test line configurations. (3) Results: The fastest assay time was 1 min, whereas even the slowest assay was within 10 min. With the passive flow approach, the limits of detection (LOD) of 0.1 and 0.5 ppm for total hazelnut protein (THP) and total peanut protein (TPP) in spiked buffer were reached, or 1 and 5 ppm of THP and TPP spiked into matrix. In comparison, the active flow approach reached LODs of 0.05 ppm for both analytes in buffer and 0.5 and 1 ppm of THP and TPP spiked into matrix. The optimized LFIA configuration reached LODs of 0.1 and 0.5 ppm of THP and TPP spiked into buffer or 0.5 ppm for both analytes spiked into matrix. The optimized LFIA was validated by testing in 20 different blank and spiked matrices. Using device-independent color space for smartphone analysis, two different smartphone models were used for the analysis of optimized assays.

## 1. Introduction

Food allergens are naturally occurring proteins present in a multitude of foods. Individuals with a food allergy are sensitized towards these proteins, and exposure to them can lead to adverse, sometimes life-threatening, health effects [1]. The majority of food allergen-related anaphylaxis in Europe can be attributed to peanut and tree nut allergens [2]. Allergies towards peanuts and tree nuts commonly co-exist, making the simultaneous detection of these problematic allergens desirable [3,4].

The only way for allergic individuals to avoid an allergic reaction is for them to stick to an avoidance diet. Such diets are largely reliant upon proper allergen labeling of food products. However, currently in the European Union (EU), only ingredients which have been intentionally incorporated into a food require labeling [5,6]. This means that allergens that are unintentionally present in food, such as via cross contamination, do not need to be declared, with all associated risks for allergic consumers. As a result, many food manufacturers use voluntary precautionary allergen labeling (PAL) (e.g., ‘may contain’ statements) in order to safeguard consumers [7].

In theory, PAL statements protect the consumer from potential allergic reactions; in reality the over-use of unregulated PAL has resulted in consumers choosing to ignore these warning statements [8]. Therefore, it is imperative to engage the public with their own food allergen analysis by developing consumer-friendly detection methods [9,10]. The cornerstones to consumer-friendly allergen detection are speed, sensitivity, ease-of-use, affordability, portability, multiplexing capability and a simple read-out system. Although some specifically consumer-oriented allergen sensors are available, such as the portable gluten and peanut sensors from NIMA, more often these biosensors are still proof-of-concept assays rather than commercial tests designed for consumers [11,12,13,14], and generally they lack multiplexing and proper validation as screening methods. A shared characteristic of novel allergen detection is the increasing trend to utilize a smartphone as an interface and readout system [9,14,15,16,17]. Using a smartphone readout improves the overall ease of result interpretation by introducing an interface that the consumer is already familiar with, alongside providing a means to wirelessly transmit results to relevant stakeholders, such as food manufacturers and restaurant personnel [18]. The Lateral Flow Immunoassay (LFIA) is widely considered the gold standard for easy-to-use, low-cost, sensitive and quick screening for food safety issues. Despite their widespread application, allergen LFIAs are often based on the analysis of a single analyte, owing to the difficulties associated with multiplexing an LFIA, including the need for careful design of test line configuration to prevent upstream detection areas from affecting downstream detection areas [19,20]. Most multiplex LFIAs for food safety focus upon the detection of low-molecular weight compounds, such as antibiotics and mycotoxins [21,22]. However, this past year has seen an increase in the development of multiplex food allergen detection LFIAs, with the development of an assay for the detection of hazelnut, ovalbumin and casein in bakery products within 10 min [23]. A further example is the multiplex, low-ppm detection of both β-lactoglobulin and β-casein, two major allergenic milk proteins, within 10 min [24].

A major drawback typically associated with LFIAs is the assay duration, which usually is 10–20 min, and is affected by mass transport limitations (MTL) and binding kinetics [25]. MTLs are caused by the fact that the target analytes need to be carried across a porous membrane, such as nitrocellulose (NC) by passive, capillary flow, and thus affect the detection speed of the assay [26]. The NC capillary flow rate is measured in the time in seconds it takes the sample front to travel 4 cm. Selection of NC based on this capillary flow rate is a compromise between assay sensitivity and assay speed with mid-speed membranes (120–150 s/4 cm) offering advantages in both areas [27]. When detection speed is not a constraint, a membrane with a slower flow rate and smaller pore size increases the available binding time between the labeled antibody–analyte and the test line antibody which can result in increased assay sensitivity [27,28,29]. In order to speed up LFIAs, in combination with NC with a good flow rate, antibodies with fast association rates towards their target should be used. Antibodies can be selected for their binding kinetics by in depth surface plasmon resonance (SPR)-based antibody screening and characterization. In this way a carbon nanoparticle-based hazelnut allergen LFIA has been developed, with a 30 s assay time, which as far as we know is a world record for allergen assay speed [30].

In order to overcome restrictions typically associated with LFIAs, a flow-through immunoassay format can be used instead [31,32]. Flow-through immunoassays are reported to offer the benefits of increased assay speeds, better sensitivities—owing to the use of larger sample volumes, excellent multiplexing capabilities and the absence of the ‘hook-effect’ [27,33,34]. The hook-effect is a phenomenon that is commonly encountered in one-step, sandwich format LFIAs. It occurs where the free analyte and the analyte which is bound to a labeled antibody compete for the limited number of binding sites available on immobilized capture antibodies, leading to a reduction in colorimetric signal and sometimes false negative results [24,35,36]. Therefore, if the correct assay working range is not determined, it could lead to consumers erroneously believing a food with a high allergen content is safe.

Flow-through assays can be prepared in different ways. Passive flow-through assays consist of LFIA materials, but in a stacked arrangement, with the membrane biofunctionalized with capture antibodies on top, and the conjugate and absorbent pads layered underneath or as flow-through enzyme-linked immunosorbent assays (ELISAs) [37,38,39]. An alternative flow-through approach is to insert a biofunctionalized membrane into a syringe filter holder, applying manual or mechanical pressure to the syringe to actively control the vertical flow of the reagents and the sample [40,41]. Although flow-through formats generally allow greater freedom in geometric assay design, they are prone to inter/intra-user variability [42].

The lack of agreed regulatory allergen thresholds has stalled the development of certified reference materials, preventing true comparisons to be made between various detection methods by different kit manufacturers and researchers [43]. Therefore, in this study, we use the same bioreagents to compare different geometrically designed, paper-based, flow-through and lateral flow immunoassay configurations for the simultaneous detection of hazelnut and peanut allergens with a smartphone readout system.

## 2. Materials and Methods

### 2.1. Reagents and Consumables

Washing buffer (WB) was composed of 5 mM borate buffer (BB) (pH 8.8) diluted from a mixture of 100 mM sodium tetraborate (VWR, Leuven, Belgium) and 100 mM boric acid (Merck, Darmstadt, Germany) and bovine serum albumin (BSA; Sigma-Aldrich, Zwijndrecht, The Netherlands) was added to a final concentration of 1% (*w*/*v*). Storage buffer (SB) consisted of 100 mM BB containing BSA to a final concentration of 1% (*w*/*v*). Running buffer (RB) was prepared by adding 1% BSA (*w*/*v*) and 0.05% Tween-20 (*v*/*v*) (Merck, Darmstadt, Germany) to 100 mM BB. Phosphate buffered saline (PBS; 0.01 M; pH 7.4) was purchased from Sigma-Aldrich (Sigma-Aldrich, Zwijndrecht, The Netherlands). All solutions were prepared with water from a MilliQ-system (MQ) (>18.2 MΩ/cm) purchased from Millipore (Burlington, MA, USA). ‘Spezial Schwartz 4’ carbon nanoparticles were purchased from Degussa AG (Frankfurt, Germany). Goat anti-mouse IgG in PBS (pH 7.6) (1.2 mg/mL; AffiniPure F(ab’)_2_ Fragment GAM IgG Fcγ) used for spraying control lines/spots was purchased from Jackson Immunoresearch Laboratories Inc. (Sanbio, Uden, The Netherlands). The hazelnut (50-6B12) and peanut (51-2A12 and 51-12D2) antibodies were developed by Wageningen Food Safety Research (WFSR), Wageningen University and Research (Wageningen, The Netherlands) according to the procedure described by Bremer et al. [44]. All antibodies were buffer exchanged from PBS (pH 7.4) into 5 mM BB (pH 8.8) using Zeba™ Spin Trap columns (Thermo Scientific; Landsmeer, The Netherlands) prior to use. Passive flow-through assays were developed from a Miriad Rapid Vertical Flow toolkit (MedMira, Halifax, NS, Canada). All active flow-through assays were developed on unbacked Whatman 0.45 µm nylon (GE Healthcare, Eindhoven, The Netherlands) 0.45 µm NC or 0.2 µm NC membranes and inserted into 13 mm Swinny syringe filter holders (Merck, Darmstadt, Germany). The assembled filter holder was attached to a 10 mL syringe (Becton-Dickinson, Utrecht, The Netherlands). Lateral flow immunoassays (LFIAs) were developed on 140 CN nitrocellulose membranes (Unisart, Sartorius, Gottinghem, Germany) secured on a plastic backing (G and L, San Jose, CA, USA) overlaid with an absorbent pad (Whatman, GE Healthcare, Eindhoven, The Netherlands). All LFIAs were heat-sealed in foil packets with silica beads and stored at room temperature until use.

### 2.2. Allergen Extraction

Currently, a drawback in allergen detection is that no certified, standardized reference materials are commercially available, and antigen standards and blank matrices need to be prepared in-house [45]. The influence of food processing on the protein conformation of allergens can affect their detectability [46], but this was not explicitly investigated in this study, as the focus was comparing the performance of the same antibodies applied in different immunoassay formats.

Extracts were made from hazelnuts, peanuts, blank flour, peanut-spiked flour (8 ppm) and 20 truly different biscuits (i.e., 20 different brands and varieties; see Supplementary Information, Table S1) free from peanuts/tree-nuts, which were supplied by project partners or purchased from local supermarkets. Raw hazelnuts and unsalted peanuts were frozen whole at −80 °C for 1 h. The frozen foods were homogenized using a commercial hand blender (Braun Turbo 600 W Food Processor, Braun, Oss, The Netherlands). A total protein extract was made by adding 10 mL PBS (pH 7.4) per gram of ground sample and incubating at room temperature for 1 h. Following incubation, extracts were centrifuged at 3220× *g* for 20 min. The extracts were then filtered through a series of low protein-binding syringe filters (5 µm > 1.2 µm > 0.45 µm), and the filtrate was aliquoted and stored at −20 °C until use. To ensure sample stability, fresh aliquots were defrosted daily for experiments, and protein concentrations were determined using the NanoDrop ND 3300 (Isogen Life Sciences, De Meern, The Netherlands) prior to use. Blank biscuits were homogenized by agitating 0.5 g in a 50 mL tube with ball bearings to a fine powder. Next, 5 mL of 100 mM borate buffer was added to the tubes and agitated for 1 min with the powdered biscuit or flour. The suspension was left at room temperature for 25 min. Afterwards, extracts were filtered through a series of low protein-binding syringe filters (5 µm > 1.2 µm > 0.45 µm), aliquoted and stored at −20 °C until use. All experiments, except for matrix experiments, were performed using total hazelnut protein (THP) and total peanut protein (TPP) spiked into running buffer. For matrix experiments, 1 µL of 1000 ppm THP and TPP extract was spiked into 999 µL (*v*/*v*) of the 20 different blank biscuit extracts.

### 2.3. Carbon Black Nanoparticle Conjugation

A 1% suspension of carbon nanoparticles (CNPs) was prepared by adding 1 mL of MQ Water to 10 mg carbon and sonicating for 10 min. The resulting 1% carbon suspension was diluted five times in 5 mM BB (pH 8.8) to obtain a 0.2% suspension, which was then sonicated for 5 min. Next, 350 µL purified hazelnut or peanut antibody solution (1 mg/mL in 5 mM BB) was added to 1 mL (to make a total volume of 1.35 mL) of 0.2% carbon suspension and stirred overnight at 4 °C. The suspension was split into approximately two equal aliquots (670 µL), and 500 µL of WB was added to each before centrifuging them for 15 min at 13,636× *g* at 4 °C. Following this, the supernatants were removed, and the pellets re-suspended in WB. This process was repeated three times. After the final wash, the supernatants were discarded, and the pellets were pooled together with 1 mL storage buffer and stored at 4 °C until use.

### 2.4. Multiplex Passive Flow-through

**Figure 1:**
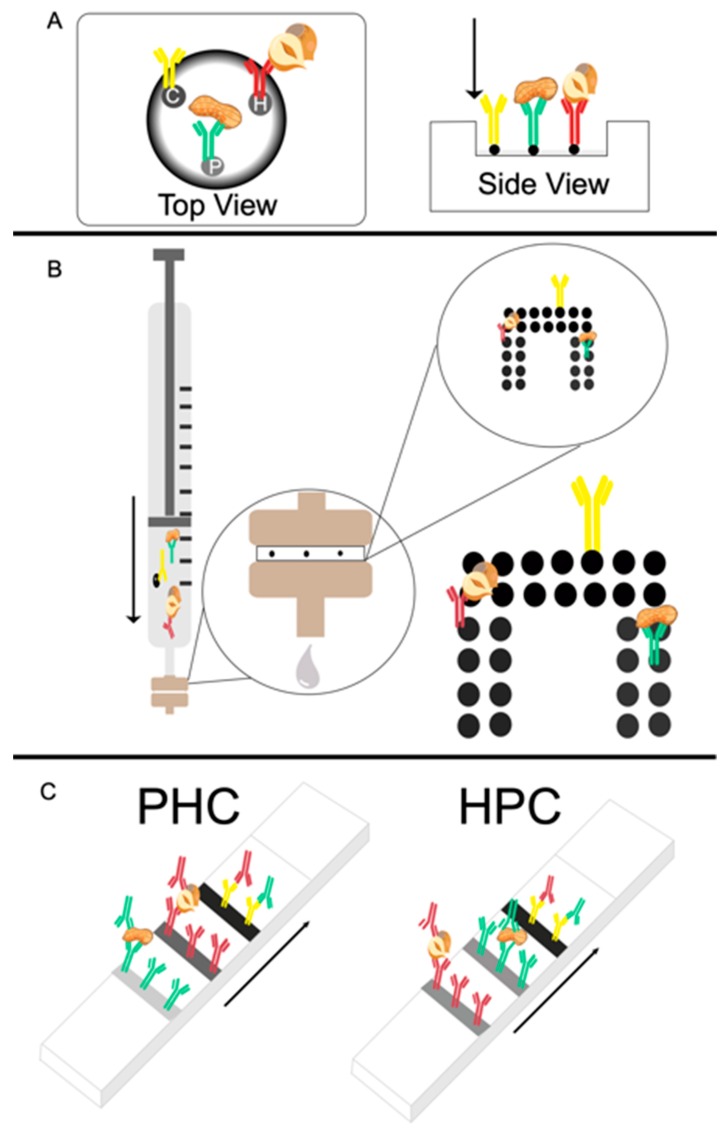
Schematic representation (not to scale) of the three flow assay formats developed. Arrows depict the flow direction and C is the control antibody (goat anti-mouse), H is the anti-hazelnut antibody and P is the anti-peanut antibody. Total hazelnut protein (THP) is indicated by the hazelnut graphic and total peanut protein (TPP) is indicated by the peanut graphic. (**A**) The passive flow assay in top-view and side-view. (**B**) The active format flow-through assay, where the syringe filter holder is enlarged, and the membrane is further enlarged to show the biofunctionalized area. (**C**) Both lateral flow immunoassay geometries as defined by the order in which sample will encounter the test and control lines: Peanut, hazelnut, control (PHC) and hazelnut, peanut, control (HPC).

The plastic cartridge, biofunctionalized membrane and absorbent pad (absorption volume of 200 µL) from a Miriad Rapid Vertical Flow technology toolkit was used to create the passive flow-through assays. A schematic representation of the passive flow-through assay is shown in Figure 1A.

The membranes were biofunctionalized by manually depositing 0.5 µL of the peanut, hazelnut and control antibody solutions (1 mg/mL) in three distinct regions using a pipette. The tip of the pipette was touched very lightly against the membrane to dispense a consistent antibody spot. The membranes were dried for 45 min. Once dried, three drops of RB were added via a dropper bottle and allowed to saturate the membrane. Immediately after, 50 µL of the mixed allergen extract (diluted in RB; 1000 ppm, 100 ppm, 10 ppm, 1 ppm, 0.1 or 0 ppm) was pipetted dropwise onto the membrane and allowed to absorb fully. Next, a 10 µL suspension of 10 × diluted carbon labeled-monoclonal antibodies (CNP-mAbs) was pipetted onto the membrane and allowed to absorb fully. Finally, three drops of RB were applied to wash the membranes. The assays were read immediately with the naked eye and an image was acquired with a smartphone camera. LOD values for visual inspection were established at the lowest concentration that reproducibly yielded a signal that could be observed and distinguished from the background by the naked eye.

### 2.5. Multiplex Active Flow-through

A schematic representation of the active flow-through assay is shown in Figure 1B. First, the most appropriate assays parameters were established including membrane type, pore size, antibody concentration for dispensing and assay conditions.

#### 2.5.1. Simplified Multiplex Flow-through

Allergen-specific antibody solutions (0.5 µL of 1 mg/mL mAb solution) and control antibody solution were manually dispensed by lightly touching the tip of the pipette to the membrane onto 0.2 or 0.45 µm pore size unbacked NC or 0.45 µm unbacked nylon membranes. The membranes were dried for 45 min and then the membranes were placed in 13 mm syringe filter holders and attached to the 10 mL syringe. The assays were performed by manually and sequentially injecting 500 µL sample (concentration series 100–0.1 ppm total protein extract diluted in RB), 1 µL of each CNP-mAb and another 300 µL of RB as a washing step. In this context, sequentially refers to the sequential loading of the syringe with sample with the CNP-mAbs on top of the sample; these were then pushed through by moving the plunger downwards in a single movement, followed by a final washing step with RB. The membranes were then removed from the filter holder, dried for 5 min, read with the naked eye and an image was acquired with a smartphone camera. 

#### 2.5.2. Multiplex Flow-through Iterative Optimization

To establish the optimum active flow-through conditions, a number of alternative assay steps were explored. The experiments aimed to reduce background staining, to improve the signal-to-noise ratio and to improve the assay sensitivity.

#### 2.5.3. Volume Optimization

Different sample and reagent volumes were tested to determine the optimum conditions for flow-through operation. Flow-through assays require larger sample volumes compared with LFIA due to reduced contact time between analyte and capture antibodies [42]. 

When using sample volumes of less than 500 µL, it was necessary to first ‘pre-wet’ the membrane with running buffer to ensure that the entire surface would be wetted. Initially, membranes were tested using 500 µL RB, followed by a 300 or 500 µL sample and 0.5 µL of each of the CNP-mAbs solutions followed by 500 µL RB as a washing step. In subsequent experiments, the volume of the CNP-mAb solution was increased to 1 µL for each CNP-mAb to maximize the signal intensity. Finally, experiments were performed using 1 mL of sample, with 1 µL of each CNP-mAb solution dispensed on top of the sample, followed by 500 µL RB.

#### 2.5.4. Pre-Mix Method

The assays were tested by pre-mixing the running buffer and CNP-labeled secondary mAbs with sample and injecting the mixture simultaneously. In this approach, 1 mL of sample, 1 mL of RB and 1 µL of each CNP-mAb were injected across the membrane, effectively causing an additional 50% dilution to the sample, when compared to the sequential method described above. The holder was then dismantled, and the membrane dried for 5 min before visual inspection.

#### 2.5.5. Filter Approach

To improve the uniform wetting of the membrane and reduce the background staining caused by the CNPs, a filter approach was tested. In this method, a 0.45 µm NC filter was placed on top of the functionalized membrane before carrying out the assay sequentially. Following the final wash step, the device was dissembled, the 0.45 µm filter carefully removed and disposed of and the membrane dried for 5 min before visual inspection.

#### 2.5.6. Aspiration Approach

To ensure sufficient wetting of the membrane, and to increase the contact time of the sample and the capture antibodies, an iterative aspiration approach was applied. In this way, when sequentially injecting the sample and CNP-mAbs, the plunger of the syringe was pumped up and down, 1, 5 or 10 times. With the increasing number of aspirations, the flux of the analyte past the membrane, and thus past the immobilized antibodies, was increased. After the final aspiration, the RB was flowed through as a washing step, the device was disassembled, and the membrane dried for 5 min before visual inspection and photographing with a smartphone camera.

#### 2.5.7. Multiplex Array Layout

The flow-through array was spotted using the XYZ 3060 BioDot Dispense Platform (Irving, CA, USA). The array was composed of 14 (2 × 7 array) control spots (0.25 mg/mL) and with each analyte having 12 (2 × 6 array) spots (0.25 mg/mL), with a drop size of 100 nL and an offset of 1 mm between each dot (see Figure 1B). The membranes were left to dry overnight prior to testing.

#### 2.5.8. Optimized Active Flow-through Operation Protocol

A 0.45 µm NC filter, acting as a vertical flow diffuser, was placed on top of the biofunctionalized membrane. The filter and membrane were then placed, biofunctionalized side up, into the syringe filter holder. A polytetrafluorothylene (PTFE) gasket was placed on top of the membrane to seal the fluid pathway, giving the assay an actual flow path of 10 mm. The syringe holder was then attached to a 10 mL Luer-Lock™ syringe. The assay was performed sequentially as described in Section 2.5.1. First, 1 mL of sample topped with 1 µL of each CNP-mAb solution was aspirated 10 times across the membrane (only THP or only TPP or mixture of both diluted in RB at 100, 10, 1, 0.1 and 0 ppm). Following this, 500 µL RB, as a washing buffer, was flowed through the membrane. Finally, the syringe filter holder was disassembled, and the membrane removed and placed on an absorbent pad for drying. To determine whether the immobilized test antibodies suffered from non-specific binding towards the other target, the assays were tested using just THP or just TPP extract spiked into RB. 

Blank buffer measurements were performed 10 times to test for false positives. The membranes were visually inspected and photographed with a smartphone camera after 5 min. LOD values for visual inspection were established at the lowest concentration that reproducibly yielded a signal that could be observed and distinguished from the background by the naked eye.

### 2.6. Multiplex Lateral Flow Immunoassay

Lateral flow immunoassays were manufactured using NC (flow rate of 140 s/4 cm) cut to approximately 4 cm length. The NC membrane was secured on a plastic backing, with 4.5 cm of absorbent pad overlapping one end of the NC. Two different test line configurations (as depicted in Figure 1C) were designed and produced using the XYZ BioDot dispensing platform. The first configuration had the control line (0.25 mg/mL) dispensed at 10 mm from the absorbent pad, the hazelnut line (0.25 mg/mL) at 5 mm from the control line and the peanut line at 5 mm from the hazelnut line, with 10 mm of blank membrane at the bottom of the strip, hereafter referred to as PHC. The second arrangement had the control line at 10 mm from the absorbent pad, the peanut line at 5 mm from the control line and the hazelnut line at 7 mm from the peanut line with 8 mm of blank membrane at the bottom of the strip, hereafter referred to as HPC.

#### Multiplex LFIA Operation Protocol

Firstly, the multiplex LFIAs were tested for non-specific binding by testing 10 × each of the LFIAs in blank running buffer (RB). The LFIAs were inserted into individual microwells of a 96-well plate containing 1 µL of each of the CNP-mAbs and 100 µL of RB (blank). The strips were left to run for 5 min. Next, the LFIAs were tested for specificity by testing in either just THP or TPP extract spiked into RB. LFIAs were placed into the individual microwells of a 96-well plate containing either just THP or TPP (1 µL) spiked into RB, in decreasing concentration with RB (99 µL) and 1 µL of each carbon-labeled mAb. The strips were left to run for 5 min before photographing with a smartphone camera. Finally, the assays were tested using the same conditions in decreasing concentrations (100, 10, 1, 0.5, 0.1 ppm) of both THP and TPP spiked in RB (in triplicate). Calibration series were tested with both formats of the LFIA using (i) 1 µL of sample (diluted in RB) and 99 µL of RB (hereafter, 1:99, sample: RB), (ii) 25 µL of sample (diluted in RB) and 75 µL of RB (hereafter, 25:75, sample: RB), and (iii) 75 µL sample (diluted in RB) and 25 µL of RB (hereafter, 75:25, sample: RB). The 75:25 sample: RB experiments were specifically designed to trigger the hook-effect to determine when the sample volume becomes the limiting factor.

The membranes were visually inspected and photographed with a smartphone camera after running for 5 min. LOD values for visual inspection were established at the lowest concentration that reproducibly yielded a signal that could be observed and distinguished from the background by the naked eye.

### 2.7. Smartphone Readout and Data Analysis

Smartphone photographs were acquired using Open Camera (version 4.0.3) and analyzed using a Huawei P20 smartphone (Huawei Technologies, Shenzen, China) according to the method developed by Ross et al. [27] using two freely downloadable apps from the Google Play Store. The red, green, blue (RGB) values were obtained for test regions of assays using the RGB Color Detector (version 1.0.58). Using the crosshair function in the app, test dots on the flow-through membrane or three distinct regions on the test line of the LFIA were selected and the color values were averaged and recorded. Background measurements were also made above and below the test areas to determine an overall background level for subtraction from results. Alternatively, results were normalized by dividing the value of each test region by the corresponding control region, as has been performed in literature [35,47,48]. Using ‘Nix Pro Color’ (version 1.31), the RGB values were converted to luminosity, A, B (LAB) values; a device-independent color space that more accurately represents how humans interpret color intensity. 

Additionally, to show the device-independent nature of LAB measurements, the optimized assays were also analyzed using a Google Pixel 2 XL smartphone (Google, Mountain View, CA, USA). The obtained values were used to plot calibration curves for L (luminosity) of the LAB values as a function of allergen concentrations spiked into RB, using Microsoft Excel. LOD values were obtained from these calibration curves by visual evaluation.

### 2.8. Matrix Experiments and Validation

To validate the assays, they were also tested in spiked food matrices. All assays were tested in a decreasing concentration of THP and TPP, spiked directly into a blank biscuit matrix extract to determine the matrix effects. Additionally, the optimized LFIA (PHC) was more extensively validated by testing in 20 truly different blank matrix extracts. In this way, LFIAs were placed in individual microwells containing 25 µL blank matrix extract (*n* = 20) and 75 µL RB and left to run for 10 min to determine whether any false positives occurred. Additionally, 1 ppm of THP and TPP was spiked into the 20 different blank matrix extracts (1 µL of 1000 ppm THP and TPP sample into 999 µL (*v*/*v*) blank matrix extract) and the LFIAs were tested using both 25 µL spiked matrix plus 75 µL RB and 1 µL spiked matrix extract plus 99 µL RB. Assays were left to develop for 10 min. Finally, the optimized LFIAs were also tested in blank flour matrix extract and spiked peanut flour matrix extract in both 25:75 and 1:99 dilutions in RB.

## 3. Results and Discussion

### 3.1. Multiplex Passive Flow-through Assay

An overview of conditions, quantitative and qualitative results for spiked buffer experiments for the passive flow-through assay, can be found in Table 1. The visual limit of detection (LOD) for the passive flow-through was established by testing in decreasing concentrations of THP and TPP extracts spiked in RB. The visual LODs were determined as 0.1 ppm and 1 ppm and smartphone LODs 1 and 10 ppm for hazelnut and peanut, respectively (*n* = 3), whereas no visible spot was obtained for blanks (see Table 1 and Supplementary Information, Figure S1A). Following the addition of the CNP-mAbs to the passive flow-through assay, the positive spots appeared within 5 s, a detection speed which is unparalleled by LFIA. Even when using the high-speed LFIA described in [30] the appearance of the positive result took 30 s, due to MTL limitations of the solution that needs to wick through the membrane before reaching test lines. Three drops of RB were added to the flow-through assay to wash the unbound CNPs from the membrane. Using dropper bottles with pre-defined drop volumes for the delivery of RB makes the assay easy to perform and means that pipettes are unnecessary. A further benefit is that the result can be directly read through the window of the cassette by the naked eye without having to disassemble the device. However, when recording a smartphone image of the membranes, these do need to be removed from the plastic cassette to avoid shadowing. Despite the washing step, the membranes had variable background staining, which made it impossible to obtain calibration curves from the images acquired with a smartphone. The reason for the appearance of background staining probably lies with the polydispersity of the CNP, which can form aggregates of several hundred nm, which are too large to be flowed through the pores. A drawback of this specific passive flow assay format is the lack of freedom in geometric assay design as bio-reagents required manual spotting by pipette. However, such a limitation could be easily overcome by biofunctionalization of the membranes before having them cut to the factory-made circular size.

### 3.2. Multiplex Active Flow-through

An overview of conditions, quantitative and qualitative results for spiked buffer experiments for the active flow-through assay can be found in Table 1. The assays using the 0.45 µm pore size nylon and NC membranes were ineffective, and no spots (including control spots) appeared on these membranes. This can be attributed to 0.45 µm being too large a pore size and the majority of the analyte and labeled antibodies passing through the membrane, which is confirmed by the dark coloration of the waste liquid when using this assay membrane. Therefore, the 0.2 µm pore size NC membrane was determined to be the most suitable for this application. 

During the optimization steps, active flow-through assays were tested using 0.5 µL of each CNP-mAb solution, but this only yielded faint detection spots. In subsequent experiments the volume of the CNP-mAb solution was increased to 1 µL of each CNP-mAb which improved the readability. Additionally, volumes of 500 µL and 1 mL of sample were tested, with the sensitivity improving with the increased sample volume, without the appearance of a hook-effect, even at high concentrations. Although in this manually spotted initial format, LODs of 0.5 and 0.1 ppm could be reached for peanut and hazelnut (see Supplementary Information, Figure S1B), respectively, false positives were also detected when testing the assays in a blank sample (1 in 5 false positives). Using a pre-mix approach did improve the overall user-friendliness of the assay, as the operator only needed to pass the liquid containing the sample, CNP-labeled mAbs and RB through once without the necessity of removing and reinserting the plunger, but this method consistently resulted in false positives in the blank samples. Contrastingly, using the sequential method increased the difficulty of the assay, but prevented false positives owing to the washing step at the end. The addition of a 0.45 µm NC filter on top of the biofunctionalized membrane increased the (smartphone) readability of the assay. Besides filtering the larger sized CNPs, reducing the level of background staining, the filter also acted as a flow diffuser. In this way, uniform wettability of the membrane was achieved, resulting in better reproducibility compared to when it was performed without the filter. Although the filter improved the readability of the membranes, it also further complicated the user-friendliness of the method, as it needed to be carefully removed from the biofunctionalized membrane before the results could be read.

The sensitivity of the assay was improved by increasing the number of sample aspirations across the membrane (see Supplementary Information, Figure S2). Flow-through assays are subject to unidirectional flow and require capture antibodies with rapid association rates in order to achieve binding or require extended sample/reagent incubation times [48]. By increasing the number of sample aspirations, the flux of the CNP-mAb-analyte complex past the immobilized antibodies, and the potential of binding, is increased. Of all the tested parameters the most appropriate assay conditions were determined to be a 0.45 µm filter on top a 0.22 µm NC membrane biofunctionalized with 0.25 mg/mL control and test spots and aspirating 1 mL of sample with 1 µL of CNP-mAb solution 10 times back and forth through the membrane. Subsequently, 500 µL of RB was injected as a washing step. Although these conditions allowed for the assay to reach very low LODs, they also meant that this method generated a high volume of chemical waste (1.5 mL), which needs to be safely disposed of.

**Figure 2:**
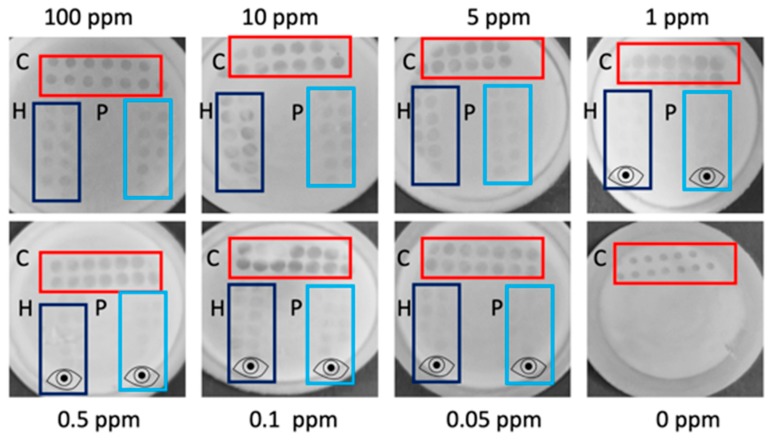
Active flow-through assay calibration range. Assays were tested in decreasing concentrations (100–0.05 ppm) of Total Hazelnut Protein (THP), Total Peanut Protein (TPP) spiked into Running Buffer (RB) and in blank RB. The control region is indicated by C and outlined in red, the hazelnut region by H and outlined in dark blue and the peanut region by P and outlined in light blue. There is an evident decrease in test dot intensity as the concentration of total protein in the sample decreases. The eye icon is used to indicate test regions that are visible to the naked eye but more difficult to read in the smartphone image. The visual limit of detection is established at 0.05 ppm for both analytes.

When testing active flow-through membranes in decreasing concentrations of THP and TPP spiked into RB, visual LODs of 0.05 ppm (*n* = 3) could be reached for both targets, an LOD which is so far un-met by commercially available allergen assays [8]. This LOD is less obvious from the smartphone image (LODs of 0.5 ppm for both THP and TTP) compared with reading by naked eye (see Figure 2). Therefore, eye symbols are inserted in Figure 2 to designate the lowest concentration that could still be read visually. Despite the active flow-through approach reaching lower LODs than the passive flow-through assay, the assay was more complicated to perform and used a far greater sample volume. 

### 3.3. Multiplex Lateral Flow Immunoassay

**Figure 3:**
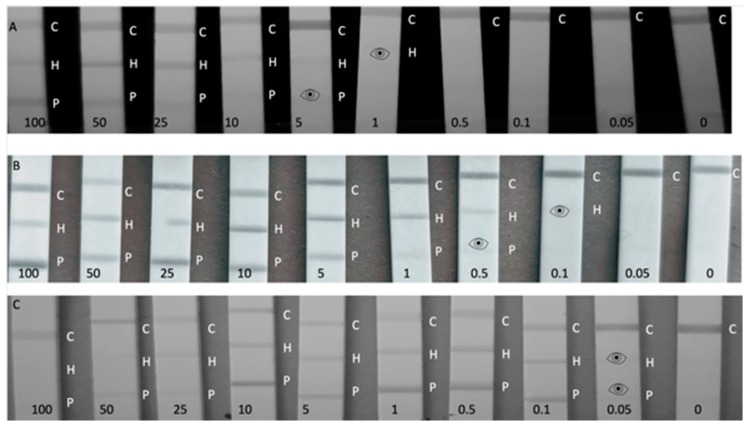
Calibration range (100–0.05 ppm) of Total Hazelnut Protein (THP), Total Peanut Protein (TPP) spiked into Running Buffer (RB) and blank RB, where the control line is indicated by C, the hazelnut test line by an H and the peanut test line by a P. A positive result can be still read with the naked eye, but is difficult to see in the smartphone image, thus an eye icon has been used to indicate the visual LOD. (**A**) Peanut, Hazelnut, Control (PHC) line configuration using 1 µL of spiked sample and 99 µL RB. (**B**) PHC using 25 µL of spiked sample and 75 µL RB. (**C**) PHC using 75 µL of spiked sample and 25 µL RB.

An overview of conditions, quantitative and qualitative results for spiked buffer experiments for the LFIAs can be found in Table 1. The LFIAs were both able to achieve single analyte detection and a true blank result every time (0% false positives at 0 ppm; *n* = 10). When testing PHC with 1 µL of sample, 1 µL of each CNP-mAb and 99 µL of RB, visual LODs of 1 and 5 ppm were achieved by the naked eye (see Figure 3A) for hazelnut and peanut, respectively, with a clear decrease in intensity in the test line with decreasing concentration of the sample. When the LFIAs have a low signal intensity, the naked eye is still superior at distinguishing between a positive or negative signal, and the lower visual LODs are indicated by the eye icon in Figure 3. However, these visual readings are performed by a trained person, and the distinction between signal and no signal at the lowest concentrations is not trivial. In comparison, when the same anti-hazelnut antibody was applied in a single-plex LFIA, an LOD of 0.1 ppm in spiked buffer was reached, which suggests that having an additional test line on the LFIA can compromise the overall sensitivity [30]. Still, the multiplex LODs are in accordance with commercially available allergen single-plex LFIAs, which report LODs within this range. However, lack of standardized, certified reference materials in the allergen industry means that each reported assay is developed using antibodies specific to different allergenic components (total soluble protein vs. allergen-specific proteins) and tested and validated using different analytes [9,45], thus underlining that true comparisons can only be made when bioreagents and samples are kept constant, as in this research. To optimize the multiplex LFIA and improve the LOD, the sample volume was increased to 25 µL (diluted in RB) in 75 µL RB. By increasing the sample volume to 25 µL (thus concentrating the sample 25 × compared with the 1 µL sample volume) LODs of 0.1 and 0.5 ppm for hazelnut and peanut were reached respectively (see Figure 3B). 

Despite the assay sensitivity improving with the increased sample volume, with these conditions at concentrations of 100 ppm and higher, a reduction of the intensity of the upper line (hazelnut) could be observed, as has been witnessed by Galan-Malo et al. [24]. Although this was not considered a false negative, as three distinct lines were still clearly visible, it did warrant further exploration into the extent of the hook-effect in more concentrated samples.

To further investigate the extent of the hook-effect and its potential to limit the upper dynamic range of the LFIA assay, the PHC format was also tested in 75 µL of sample extract diluted with 25 µL RB (see Figure 3C). These conditions resulted in a more pronounced hook-effect with LFIAs tested at 1000 ppm appearing to be false negatives, and at 100–50 ppm exhibiting decreased test line signals. As well as just testing high analyte concentrations, it is important to test different sample-to-RB ratios, as increasing sample volume has a noteworthy influence on the appearance of the hook-effect. In order to avoid the hook-effect it is imperative to use the correct volume of diluted sample. Despite this, PHC in the 75:25 conditions did achieve a lower LOD of 0.05 ppm for both analytes in RB. Therefore, PHC could still be used with 75:25 conditions for testing trace allergen levels, so long as the sample is also tested in the 1:99 and 25:75 conditions to ensure no false negatives arise at high concentrations. The optimum conditions from PHC were determined to be 25:75. When testing HPC in the 1:99 conditions, LODs of 5 and 1 ppm (see Supplementary Information, Figure S3) were reached for peanut and hazelnut, respectively, with the LODs decreasing to 1 and 0.1 with the 25:75 arrangement. But for HPC, the hook-effect was greater in 25:75 compared with PHC with concentrations of 100 and 50 ppm experiencing reduced intensity on both the control and the peanut lines, complicating quantitative analysis. The larger hook-effect in this configuration could be because the upstream (hazelnut) test line comes into contact with the sample first, and this mAb has a rapid association rate and high affinity for THP, and so it becomes quickly saturated [30].

So, the optimum condition for HPC was the 1:99 protocol, although this was significantly less sensitive compared with the optimized PHC assay. For this reason, PHC was determined to be the optimum test line configuration with the best working conditions being 25:75 in the working range of 100–0.1 ppm. Therefore, PHC was used for further smartphone quantification and validation experiments.

### 3.4. Smartphone Readout and Analysis

**Figure 4:**
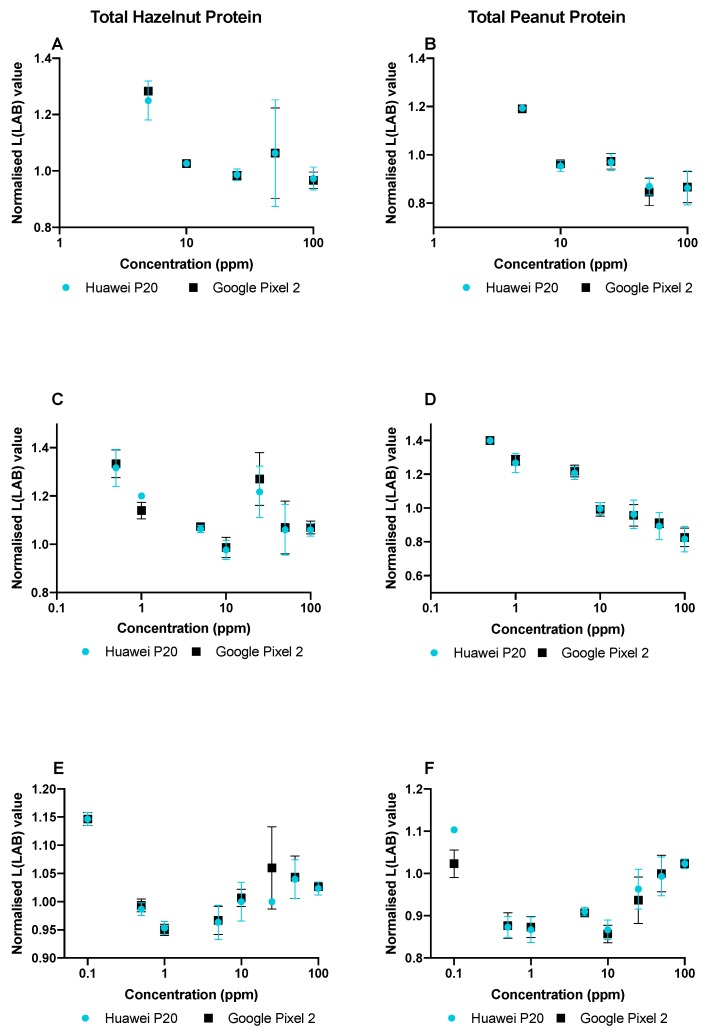
Smartphone calibration curves for the normalized (L) LAB values of the test lines of a Peanut Hazelnut Control (PHC) assay as a function of the concentration of Total Hazelnut Protein (THP), and Total Peanut Protein (TPP) (100–0.1 ppm) tested using two different smartphone models. All calibration ranges were performed in triplicate in spiked Running Buffer (RB). All L(LAB) values have been normalized by dividing the test line values by the control line values. (**A**) Hazelnut tested in 1 µL of sample in 99 µL of running buffer (RB) (**B**) Peanut tested in 1 µL of sample in 99 µL of RB. (**C**) Hazelnut tested in 25 µL sample in 75 µL of RB. (**D**) Peanut tested in 25 µL sample in 75 µL of RB. (**E**) Hazelnut tested in 75 µL sample in 25 µL of RB. (**F**) Peanut tested in 75 µL of sample in 25 µL of RB. Error bars show standard deviation (SD) from triplicate measurements.

Smartphones are ever-increasing in popularity for analyzing colorimetric assays. Most often, smartphone analysis is based on specific apps which relate a particular color intensity to a certain concentration of analyte. In the absence of a specific app, it has been shown by Ross et al. [30] that it is possible to use freely downloadable apps from the Google Play Store to analyze endpoint, smartphone image color intensity values. By converting RGB values to LAB values, luminosity or intensity can be plotted as a function of concentration in a calibration curve. In sandwich immunoassay formats with CNP labels, a higher L value corresponds to a lower analyte concentration. As LAB color space is device-independent, the same results can be potentially achieved using different smartphone models. For analysis of PHC and HPC (in triplicate) the normalization of the (L)LAB values was carried out by dividing the L values of the test lines by the L values of the control lines. The method of dividing the test line response by the control line response (T/C ratio) is a technique commonly used for the quantification of sandwich LFIAs [35,47,48,49]. The results for PHC can be found in Figure 4, and the HPC smartphone calibration curve can be found in Figure S4 in Supplementary Information.

Two smartphone models were used for the device independent LAB analysis of PHC assays (in RB in triplicate), as can be seen in Figure 4 where A, C and E show the curves for THP in 1:99, 25:75 and 75:25 (sample: RB) and B, D and F show the curves for TPP in 1:99, 25:75 and 75:25 (sample: RB). A higher normalized L value was obtained for hazelnut at 25–100 ppm using the 25:75 conditions, as can be seen in Figure 4C. Comparatively, peanut did not appear to be subject to the hook-effect under 25:75. Using 75:25 conditions (see Figure 4E), concentrations of 50 and 100 ppm resulted in a higher normalized L value for hazelnut (i.e., weaker signal). Furthermore, under these conditions the hazelnut T/C ratio for 10 ppm and 25 ppm gave the same normalized L value, highlighting that the hook-effect was still evident, even at these lower concentrations. Comparatively, peanut in 75:25 (see Figure 4F) gave higher normalized L values at concentrations of 25–100 ppm, again indicating with increasing sample volume and concentration the likelihood of the hook-effect being increased. The only crucial variation between the two smartphone measurements using the different models was obtained for the peanut line using 75:25 at 0.1 ppm (see Figure 4F). However, this is the smartphone LOD, and detection spots were already more difficult to read. As well as this, the current method relies on manually selecting regions of interest on the control and test lines, rather than being able to read the values across the whole line. Therefore, please note that the results also include any errors due to not selecting the exact same regions, and this can also cause variations in the obtained color values. 

Additionally, to compare different smartphone quantification methods, all smartphone readable assays were also analyzed by making a background subtraction as can be seen in Supplementary Information (see Figures S5 and S6). However, when analyzing the LFIAs in this way the differences in background readings, due to discrepancies in lighting conditions caused by recording an image of the entire calibration range simultaneously under ambient lighting conditions, meant that a simple background subtraction was insufficient. However, for active flow-through assays the background subtraction was found to be the most effective analysis method (see Supplementary Information, Figure S6A), whereas the T/C method resulted in larger standard deviations (see Supplementary Information, Figure S6B). This could be attributed to the membranes being photographed independently, so the small membranes were subject to the same ambient lighting conditions and did not have such variable background readings. By using two data processing methods it is evident that the selected data processing method plays a crucial role for the quality of the semi-quantitative information that can be obtained from raw results. 

### 3.5. Matrix Experiments and Validation

To determine their applicability to real life samples, the assays were tested using THP and TPP spiked into blank biscuit matrix extracts. The passive flow-through format was able to achieve visual LODs of 5 and 1 ppm for peanut and hazelnut. These LODs are higher than previously observed in spiked buffer experiments, showing that the matrix extract did have some influence on the detection of the analytes. When testing in this way, the passive flow membranes had greater background staining compared with in spiked buffer experiments. This can be attributed to the overall reduction of reagents, BSA and tween-20 in the assay buffer, as the sample was spiked into a matrix extract rather than into the RB.

In comparison, the active flow-through membranes did not suffer with increased background staining due to the use of the additional filter on top of the membrane and subsequent washing step. The active-flow assay reached visual LODs of 0.5 and 1 ppm for THP and TPP in spiked matrix extract, however the intensity of the detection spots was fainter compared with spiked buffer samples because of the reduction of buffer reagents responsible for good flow. 

Therefore, whilst visual readout was possible, the construction of calibration curves based on smartphone images could not be achieved.

**Figure 5:**
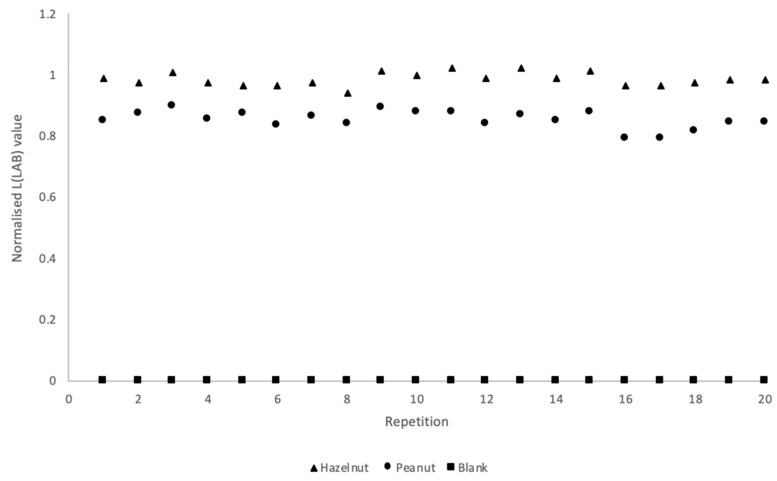
Smartphone validation of Peanut Hazelnut Control (PHC) assay using 20 truly different blank biscuit samples (square markers) and 20 truly different biscuit samples spiked at the screening target concentration of 1 ppm Total Hazelnut Protein (THP), and total peanut protein (TPP). Normalized L (LAB) values were obtained by dividing the test line response by the corresponding control line response.

PHC was tested in both 25:75 and 1:99 of spiked matrix in RB to determine the visual LOD in matrix extract, as can be seen in Supplementary Information Figure S7. When using 25 µL sample (THP and TPP spiked into matrix extract) and 75 µL RB a LOD of 0.5 ppm could be reached for both analytes (see Supplementary Information, Figure S7A). At higher concentrations (100 ppm +) there was decreased intensity for the hazelnut line. This can be attributed to the hook-effect. For the spiked matrix extract experiments, the PHC assays were run for 10 min, due to the reduction of reagents BSA and tween-20 from spiking sample into matrix extract rather than RB, affecting the flow of the sample. Additionally, PHC was tested in 1 µL of spiked matrix extract:99 µL of RB (see Supplementary Information, Figure S7B). Visual LODs of 10 and 5 ppm were reached for peanut and hazelnut, respectively. The PHC assay was fully validated using 25:75 conditions by evaluating 20 truly different blank matrices and determining that no false positives occurred. Additionally, the 20 blank matrices were spiked with 1 ppm THP and TPP. In the absence of agreed regulatory levels for food allergens, a screening target concentration (STC), based on VITAL 2.0 levels of 1 ppm, was selected [8,50]. The LFIAs were able to detect the allergens with both visual and smartphone readout at 1 ppm in all 20 samples, as can be seen in Figure 5 and as is summarized in Table 2. The excellent reproducibility at the STC level clearly suggests that a simple device-independent smartphone readout may provide semi-quantitative data.

Finally, to confirm the capability of the optimized LFIA in detecting allergens in raw ingredients, blank flour and peanut-spiked flour samples were briefly tested. The LFIAs correctly did not detect either of the allergens in the blank flour (*n* = 4). Furthermore, PHC specifically detected only peanut in the peanut-spiked flour (*n* = 4) with no false hazelnut positives being observed. The detection of peanut was not adversely affected by using the accelerated 30 min extraction procedure for the spiked flour. Further developments should include simplified and faster extraction methods.

## 4. Conclusions

Quick and accurate detection of food allergens is of critical importance for food safety; it is particularly relevant if such testing procedures can be easily performed by the consumer, and therefore, there is an evident requirement for simple and robust testing procedures. Two formats of multiplex flow-through immunoassays have been developed and compared with two test line configurations of LFIA, all developed using the same bioreagents and against the same targets in order to allow a true comparison.

Two recent review papers have extensively outlined commercially-available and proof-of-concept single-plex and multiplex allergen immunoassays and biosensors, and the assays reported in this study have matched or surpassed these previously-reported LODs [9,51]. All the developed multiplex assays were able to detect both analytes in the low ppm range within minutes. It is important to note here that our screening concentrations always related to total protein extracts from either peanuts or hazelnut, and therefore, the concentration of specific allergenic proteins is expected to be even lower than the reported values. This in turn means that the reported LODs are underestimating the true sensitivity of the immunoassays in this work. The passive flow-through format offered a way to rapidly develop a fast flow-through assay. However, this specific format was limited by the need to manually biofunctionalize the membranes, limiting their reproducibility. The active flow-through assay could achieve very low limits of detection with no false negatives when following the optimization steps. However, it is these optimization steps that made the assay more complicated to perform for a non-expert user such as a consumer. In future versions, the use of a mechanical pump could improve the user-friendliness, although this would introduce an additional and costly element into the procedure, limiting the portability of the assay. It should be reiterated that the assays within this study were performed by a trained scientist, and the active flow-through method is not recommended for untrained users. In comparison, the LFIAs, when using the optimized assay conditions for each configuration, resulted in no false positives. However, outside of working conditions, both configurations of LFIA did experience a hook-effect at high concentrations, a phenomenon commonly encountered in sandwich LFIA, where a falsely low signal occurs at high analyte concentrations. As the hook-effect is concentration-dependent, it can be avoided/limited by assay optimization.

To demonstrate their applicability to real life bakery products and raw ingredients, all assays were tested in decreasing concentrations of analyte spiked into the matrix extract. Additionally, the PHC assay was validated as a screening method in spiked matrix extract, blank matrix extract (*n* = 20) and incurred spiked flour, proving its capability of detecting the target even in complex matrices. The majority of commercially-available allergen detection LFIA test kits can detect a single analyte at 1–10 ppm [9,51]. Comparatively, PHC was able to detect both analytes at 0.5 ppm of THP and TPP spiked into a blank biscuit matrix extract, affirming its place as one of the most sensitive allergen LFIAs. This LOD was in agreement with the LOD using the same hazelnut antibody in a previously-reported single-plex assay [30]. Finally, all assays were (semi-)quantified by smartphone readout. At this stage no additional external equipment was used for the image recording, so the LFIA membranes were subject to ambient lighting conditions. To compensate for the lighting conditions a normalization factor (T/C ratio) was applied. By using device-independent (L)LAB values, it was possible to obtain comparable results using two distinct smartphone models. The ability to use different smartphone models for reading the same assays is a characteristic that is highly desirable, but not often reported, within smartphone analysis. In future developments, researchers should focus on improving the ease of use of these assays by integrating sample preparation, limiting the user interaction with the assay, as well as by developing a consumer-friendly app as a user interface which can directly analyze data with minimal user input.

## Figures and Tables

**Table 1:** Comparison of optimized Flow-through and Lateral Flow parameters (RB *).

Parameter	Passive Flow-through	Active Flow-through	PHC **	HPC **
**Visual/Smartphone LOD (ppm);** **Hazelnut (h)** **Peanut (p)**	h: 0.1/1 p: 1/10	h: 0.05/0.5 p: 0.05/0.5	h: 0.1/0.5 p: 0.5/0.5	h: 1/5 p: 5/10
**Working Range (ppm)**	1000–0.1	1000–0.05	100–0.1	10,000–0.1
**Assay Duration (total assay time, incl. drying)**	5 min	10 min	5 min	5 min
**Time until result appearance**	5 s	5 s	30 s–1 min	1 min
**Sample extracted volume (µL)**	50	1000	25	1
**Flexibility of multiplexing**	Low—requires manual dispensing bioreagents	High—Printing nL/µL size dots or multi-line	Medium—test line configuration and positioning of antibodies has an influence.	Medium—test line configuration and positioning of antibodies has an influence.
**Non-Expert Ease of Use**	Easy	Challenging	Easy	Easy
**False positives in blank RB** (*n* = number of tested samples within assay working range)	Y (*n* = 3)	Y (*n* = 10)	N (*n* = 10)	N (*n* = 10)
**False negatives in spiked RB** (*n* = number of tested samples within assay working range)	N (*n* = 3)	N (*n* = 21)	N (*n* = 24)	N (*n* = 18)
**Equipment used**	Assay cassettes, dropper bottle, pipette	10 mL syringe, syringe filter holder, assay membrane, additional filter, pipette, waste beaker	LFIA, pipettes, microwell plate	LFIA, pipettes, microwell plate
**Waste**	High plastic consumption (cassettes)	High plastic consumption (syringes) + need for disposal of high volumes liquid waste	Nitrocellulose strips and well plate + disposal of small volume liquid waste	Nitrocellulose strips and well plate + disposal of small volume liquid waste

* All measurements were made using total hazelnut protein and total peanut protein (THP and TPP) spiked into running buffer (RB). ** Where the peanut, hazelnut, control geometry is defined by PHC and the hazelnut, peanut, control geometry is defined by HPC.

**Table 2:** Matrix experiments for the optimized PHC assay, all measurements made in spiked matrix extract.

Parameter	PHC * (Matrix Extract)
LOD	0.5 ppm both analytes
Working range	100–0.5 ppm
Assay duration (total assay time incl. drying)	10 min
Time to result	1.5–2 min
Sample volume	25 µL
Reproducibility ** (*n* = 20)	Hazelnut: 2.5%
	Peanut: 3.4%
False positives (*n* = 20)	0
False negatives (*n* = 20)	0

* PHC = Peanut, hazelnut, control geometry lateral flow immunoassay. ** Reproducibility defined as Relative Standard Deviation (RSD) × 100% 1 ppm of Total Hazelnut Protein (THP), Total Peanut Protein (TPP) spiked into blank biscuit matrix extract (*n* = 20). Data based on normalized L (LAB) values.

## Supplementary Materials

The following are available online at https://www.mdpi.com/2079-6374/9/4/143/s1, Figure S1: Calibration range for multiplex flow-through assays using passive and active flow. Figure S2: Calibration range for multiplex flow-through assay optimization: Sample aspirations. Figure S3: Calibration range for lateral flow immunoassay with test line configuration: Hazelnut, peanut, control (HPC). Figure S4: Calibration curve for smartphone analysis of hazelnut, peanut, control (HPC) lateral flow immunoassay. Figure S5: Calibration curve for smartphone analysis of lateral flow immunoassays using background subtraction. Figure S6: Calibration curve for smartphone analysis of active flow-through immunoassay. Figure S7: Optimized lateral flow immunoassay calibration curve in spiked matrix extract. Table S1: Ingredient and allergen information for the 20 varieties of biscuit used for matrix experiments.

## References

[1] O’Keefe A.W., De Schryver S., Mill J., Mill C., Dery A., Ben-Shoshan M. (2014). Diagnosis and management of food allergies: New and emerging options: A systematic review. J. Asthma Allergy.

[2] Weinberger T., Sicherer S. (2018). Current perspectives on tree nut allergy: A review. J. Asthma Allergy.

[3] McWilliam V., Koplin J., Lodge C., Tang M., Dharmage S., Allen K. (2015). The Prevalence of Tree Nut Allergy: A Systematic Review. Curr. Allergy Asthma Rep..

[4] Maloney J.M., Rudengren M., Ahlstedt S., Bock S.A., Sampson H.A. (2008). The use of serum-specific IgE measurements for the diagnosis of peanut, tree nut, and seed allergy. J. Allergy Clin. Immunol..

[5] European Commission (2003). Directive, EC 2003/89/EC of the European Parliament and of the Council of 10 November 2003 Amending Directive 2000/13/EC as Regards Indication of the Ingredients Present in Foodstuffs. OJEU.

[6] European Union (2011). Regulation (EU) No 1169/2011 of the European Parliament and of the Council of 25 October 2011 on the provision of food information to consumers, amending Regulations (EC) No 1924/2006 and (EC) No 1925/2006 of the European Parliament and of the Council. OJEU.

[7] Allen K.J., Turner P.J., Pawankar R., Taylor S., Sicherer S., Lack G., Rosario N., Ebisawa M., Wong G., Mills E.N.C. (2014). Precautionary labelling of foods for allergen content: Are we ready for a global framework?. World Allergy Organ. J..

[8] Soon J.M., Manning L. (2017). May contain allergen statements: Facilitating or frustrating consumers?. J. Consum. Policy.

[9] Ross G.M., Bremer M.G., Nielen M.W. (2018). Consumer-friendly food allergen detection: Moving towards smartphone-based immunoassays. Anal. Bioanal. Chem..

[10] Choi J.R., Yong K.W., Choi J.Y., Cowie A.C. (2019). Emerging Point-of-care Technologies for Food Safety Analysis. Sensors.

[11] Zhang J., Portela S.B., Horrell J.B., Leung A., Weitmann D.R., Artiuch J.B., Wilson S.M., Cipriani M., Slakey L.K., Burt A.M. (2019). An integrated, accurate, rapid, and economical handheld consumer gluten detector. Food Chem..

[12] Taylor S.L., Nordlee J.A., Jayasena S., Baumert J.L. (2018). Evaluation of a handheld gluten detection device. J. Food Prot..

[13] Alves R.C., Barroso M.F., González-García M.B., Oliveira M.B.P., Delerue-Matos C. (2016). New Trends in Food Allergens Detection: Toward Biosensing Strategies. Crit. Rev. Food Sci. Nutr..

[14] Rateni G., Dario P., Cavallo F. (2017). Smartphone-Based Food Diagnostic Technologies: A Review. Sensors.

[15] Lin H.-Y., Huang C.-H., Park J., Pathania D., Castro C.M., Fasano A., Weissleder R., Lee H. (2017). Integrated Magneto-Chemical Sensor for On-Site Food Allergen Detection. ACS Nano.

[16] Coskun A.F., Wong J., Khodadadi D., Nagi R., Tey A., Ozcan A. (2013). A personalized food allergen testing platform on a cellphone. Lab Chip.

[17] Zhang D., Liu Q. (2016). Biosensors and bioelectronics on smartphone for portable biochemical detection. Biosens. Bioelectron..

[18] Morón M.J., Luque R., Casilari E. (2014). On the capability of smartphones to perform as communication gateways in medical wireless personal area networks. Sensors.

[19] Gantelius J., Bass T., Sjöberg R., Nilsson P., Andersson-Svahn H. (2011). A lateral flow protein microarray for rapid and sensitive antibody assays. Int. J. Mol. Sci..

[20] Anfossi L., Di Nardo F., Cavalera S., Giovannoli C., Baggiani C. (2018). Multiplex Lateral Flow Immunoassay: An Overview of Strategies towards High-throughput Point-of-Need Testing. Biosensors.

[21] Peng J., Wang Y., Liu L., Kuang H., Li A., Xu C. (2016). Multiplex lateral flow immunoassay for five antibiotics detection based on gold nanoparticle aggregations. RSC Adv..

[22] Song S., Liu N., Zhao Z., Njumbe Ediage E., Wu S., Sun C., De Saeger S., Wu A. (2014). Multiplex Lateral Flow Immunoassay for Mycotoxin Determination. Anal. Chem..

[23] Anfossi L., Di Nardo F., Russo A., Cavalera S., Giovannoli C., Spano G., Baumgartner S., Lauter K., Baggiani C. (2018). Silver and gold nanoparticles as multi-chromatic lateral flow assay probes for the detection of food allergens. Anal. Bioanal. Chem..

[24] Galan-Malo P., Pellicer S., Pérez M.D., Sánchez L., Razquin P., Mata L. (2019). Development of a novel duplex lateral flow test for simultaneous detection of casein and β-lactoglobulin in food. Food Chem..

[25] Cho D.G., Yoo H., Lee H., Choi Y.K., Lee M., Ahn D.J., Hong S. (2018). High-Speed Lateral Flow Strategy for a Fast Biosensing with an Improved Selectivity and Binding Affinity. Sensors.

[26] Zhao M., Wang X., Nolte D. (2010). Mass-transport limitations in spot-based microarrays. Biomed Opt. Express.

[27] Chen P., Gates-Hollingsworth M., Pandit S., Park A., Montgomery D., AuCoin D., Gu J., Zenhausern F. (2019). Paper-based Vertical Flow Immunoassay (VFI) for detection of bio-threat pathogens. Talanta.

[28] Bishop J.D., Hsieh H.V., Gasperino D.J., Weigl B.H. (2019). Sensitivity enhancement in lateral flow assays: A systems perspective. Lab Chip.

[29] Katis I.N., He P.J., Eason R.W., Sones C.L. (2018). Improved sensitivity and limit-of-detection of lateral flow devices using spatial constrictions of the flow-path. Biosens. Bioelectron..

[30] Ross G., Bremer M.G., Wichers J.H., Van Amerongen A., Nielen M.W. (2018). Rapid Antibody Selection Using Surface Plasmon Resonance for High-Speed and Sensitive Hazelnut Lateral Flow Prototypes. Biosensors.

[31] Ekins R., Wild D. (2013). Chapter 2.5—Ambient analyte assay. The Immunoassay Handbook.

[32] Yetisen A.K., Akram M.S., Lowe C.R. (2013). Paper-based microfluidic point-of-care diagnostic devices. Lab Chip.

[33] Oh Y.K., Joung H.-A., Kim S., Kim M.-G. (2013). Vertical flow immunoassay (VFA) biosensor for a rapid one-step immunoassay. Lab Chip.

[34] Reuterswärd P., Gantelius J., Andersson Svahn H. (2015). An 8 min colorimetric paper-based reverse phase vertical flow serum microarray for screening of hyper IgE syndrome. Analyst.

[35] Rey E.G., O’Dell D., Mehta S., Erickson D. (2017). Mitigating the Hook Effect in Lateral Flow Sandwich Immunoassays Using Real-Time Reaction Kinetics. Anal. Chem..

[36] Tate J., Ward G. (2004). Interferences in immunoassay. Clin. Biochem. Rev..

[37] Clarke O.J.R., Goodall B.L., Hui H.P., Vats N., Brosseau C.L. (2017). Development of a SERS-Based Rapid Vertical Flow Assay for Point-of-Care Diagnostics. Anal. Chem..

[38] Eltzov E., Marks R.S. (2017). Colorimetric stack pad immunoassay for bacterial identification. Biosens. Bioelectron..

[39] Samsonova J.V., Safronova V.A., Osipov A.P. (2018). Rapid flow-through enzyme immunoassay of progesterone in whole cows’ milk. Anal. Biochem..

[40] Chinnasamy T., Segerink L.I., Nystrand M., Gantelius J., Andersson Svahn H. (2014). Point-of-Care Vertical Flow Allergen Microarray Assay: Proof of Concept. Clin. Chem..

[41] Burmistrova N.A., Rusanova T.Y., Yurasov N.A., Goryacheva I.Y., De Saeger S. (2014). Multi-detection of mycotoxins by membrane based flow-through immunoassay. Food Control.

[42] Joung H.-A., Ballard Z.S., Ma A., Tseng D.K., Teshome H., Burakowski S., Garner O.B., Di Carlo D., Ozcan A. (2019). Paper-based multiplexed vertical flow assay for point-of-care testing. Lab Chip.

[43] Reese I., Holzhauser T., Schnadt S., Dölle S., Kleine-Tebbe J., Raithel M., Worm M., Zuberbier T., Vieths S. (2015). Allergen and allergy risk assessment, allergen management, and gaps in the European Food Information Regulation (FIR). Allergo J. Int..

[44] Bremer M.G.E.G., Smits N.G.E., Haasnoot W. (2009). Biosensor immunoassay for traces of hazelnut protein in olive oil. Anal. Bioanal. Chem..

[45] Walker M.J., Burns D.T., Elliot C.T., Gowland M.H., Mills C.E.N. (2016). Is food allergen analysis flawed? Health and supply chain risks and a proposed framework to address urgent analytical needs. Analyst.

[46] Croote D., Quake S.R. (2016). Food allergen detection by mass spectrometry: The role of systems biology. NPJ Syst. Biol. Appl..

[47] Zhao Y., Wang H., Zhang P., Sun C., Wang X., Wang X., Yang R., Wang C., Zhou L. (2016). Rapid multiplex detection of 10 foodborne pathogens with an up-converting phosphor technology-based 10-channel lateral flow assay. Sci. Rep..

[48] Anfossi L., Di Nardo F., Giovannoli C., Passini C., Baggiani C. (2013). Increased sensitivity of lateral flow immunoassay for ochratoxin A through silver enhancement. Anal. Bioanal. Chem..

[49] Raeisossadati M.J., Danesh N.M., Borna F., Gholamzad M., Ramezani M., Abnous K., Taghdisi S.M. (2016). Lateral flow based immunobiosensors for detection of food contaminants. Biosens. Bioelectron..

[50] Taylor S.B., Christensen G., Grinter K., Sherlock R., Warren L. (2018). The Allergen Bureau VITAL Program. J. AOAC Int..

[51] Tsagkaris A.S., Nelis J.L.D., Ross G.M.S., Jafari S., Guercetti J., Kopper K., Zhao Y., Rafferty K., Salvador J.P., Migliorelli D. (2019). Critical assessment of recent trends related to screening and confirmatory analytical methods for selected food contaminants and allergens. TrAC Trends Anal. Chem..

