# Supplementary material for: A Critical Comparison between Flow-through and Lateral Flow Immunoassay Formats for Visual and Smartphone-Based Multiplex Allergen Detection
Source: Biosensors (Basel). 2019 Dec 12;9(4):143. doi: 10.3390/bios9040143 (PMC6956089; doi:10.3390/bios9040143)
Supplement: Supplementary file 1 [file biosensors-09-00143-s001.pdf]

biosensors

MDPI

Supplementary Information

A Critical Comparison between Flow-through and Lateral Flow Immunoassay Formats for Visual and Smartphone-based Multiplex Allergen Detection

Georgina M.S. Ross [1,*], Gert IJ. Salentijn [1,2] and Michel W.F. Nielen [1,2]

[1] Wageningen Food Safety Research, Wageningen University & Research, P.O Box 230, 6700 AE Wageningen, The Netherlands
[2] Wageningen University, Laboratory of Organic Chemistry, Helix Building 124, Stippeneng 4, 6708 WE Wageningen, The Netherlands
* georgina.ross@wur.nl

Table of Contents

Figure S1: Calibration range for multiplex flow-through assays using passive and active flow…..2
Figure S2: Calibration range for multiplex flow-through assay optimization: sample aspirations..2
Figure S3: Calibration range for lateral flow immunoassay with test line configuration: hazelnut, peanut, control (HPC)...3
Figure S4: Calibration range for smartphone analysis of hazelnut, peanut, control (HPC) lateral flow immunoassay...3
Figure S5: Calibration curves for smartphone analysis of lateral flow immunoassays using background subtraction 4
Figure S6: Calibration curves for smartphone analysis of active flow-through immunoassay...5
Figure S7: Optimized lateral flow immunoassay calibration curve in spiked matrix extract 6
Table S1: Ingredient and allergen information for the 20 varieties of biscuit used for matrix experiments…………………………………………………………………………………….…....7

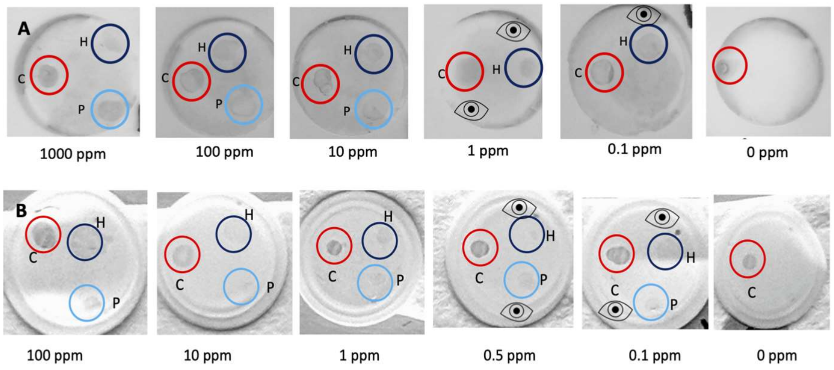

Figure S1. Calibration range for multiplex flow-through assays using (**A**) passive and (**B**) active flow. For control outlined in red (**C**), hazelnut outlined in dark blue (**H**) and peanut outlined in light blue (**P**). Assays tested in Total Hazelnut Protein (THP) and Total Peanut Protein (TPP) spiked into Running Buffer (RB; 0–1000 ppm in A and 0–100 ppm in B). Membranes were manually spotted with 0.5 µL of primary antibody (1 mg/mL). Eye icons are used to denote the lowest concentration of TPP or THP the assays are readable by naked eye, but difficult to capture on a smartphone camera.

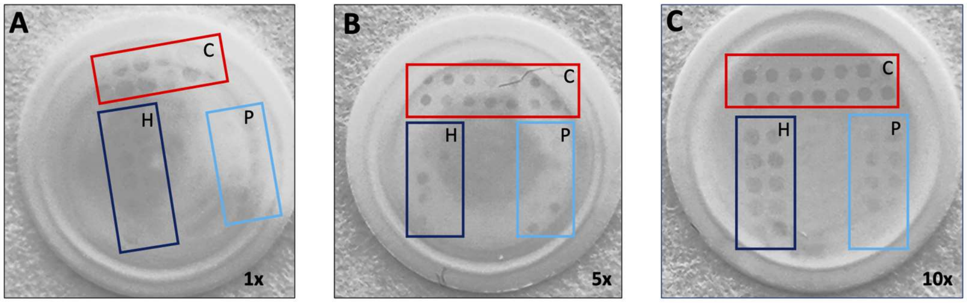

Figure S2. Calibration range for multiplex flow-through assay optimization: sample aspirations. Active flow-through assays tested in 10 ppm Total Hazelnut Protein (THP) and Total Peanut Protein (TPP) spiked into Running Buffer (RB) with 1 (**A**), 5 (**B**) and 10 (**C**) aspirations. The control region is outlined in red (**C**), the hazelnut region in dark blue (**H**) and the peanut region in light blue (**P**). There is an increase in surface wetting with the increasing number of aspirations.

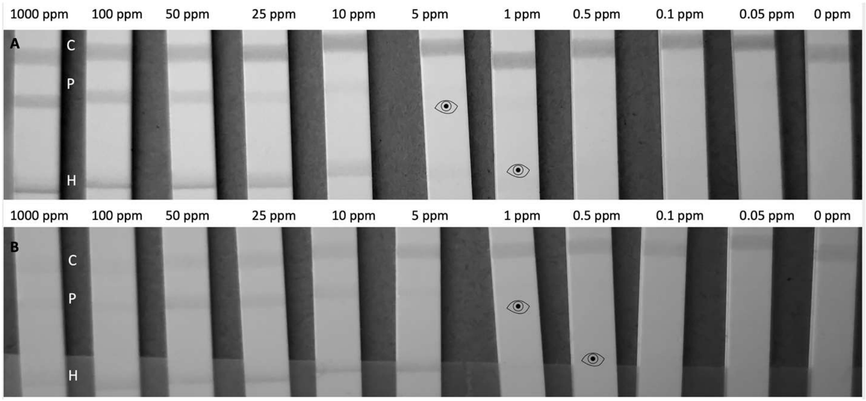

Figure S3. Calibration range for lateral flow immunoassay with test line configuration: hazelnut, peanut, control (HPC). The strips were tested with Total Hazelnut Protein (THP) and Total Peanut Protein (TPP) spiked into the Running Buffer (RB) in decreasing concentration. Where C represents the control line, P the peanut line and H the hazelnut line, and the eye icon represents the visual LOD,

which is not as clearly readable in the smartphone image. **(A)** tested in 1 µL of sample in 99 µL of RB **(B)** tested in 25 µL of sample in 75 µL of RB.

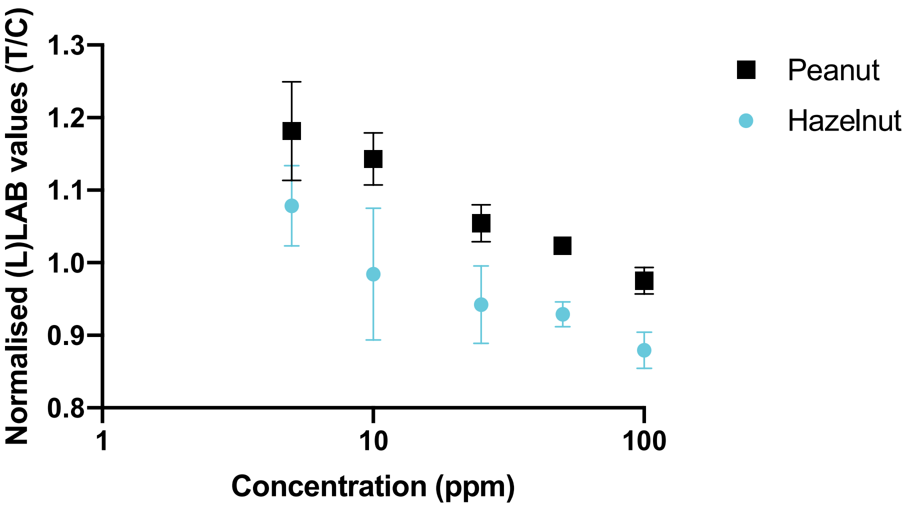

Figure S4. Calibration range for smartphone analysis of hazelnut, peanut, control (HPC) lateral flow immunoassay. Smartphone (Huawei P20) analysis, where normalized L (LAB) values (test line intensity/control line intensity; T/C) are plotted as a function of the concentration (100–5 ppm) Total Hazelnut Protein (THP) and Total Peanut Protein (TPP) spiked into Running Buffer (RB) (1 µL of the given dilution was diluted in 99 µL of RB). Error bars represent the standard deviation (n = 3). For experimental details, see the full manuscript.

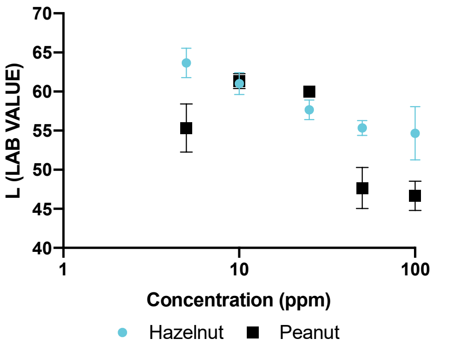

(**A**)

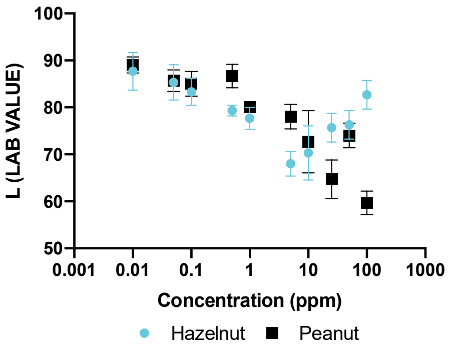

(**B**)

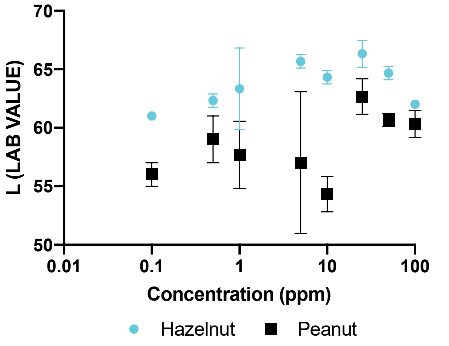

(**C**)

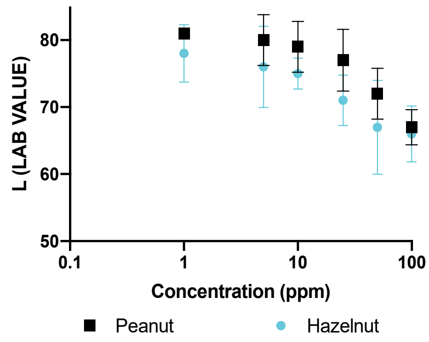

(**D**)

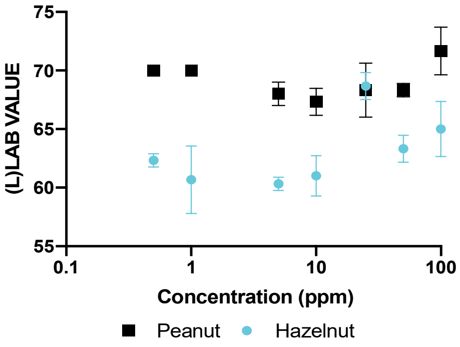

(**E**)

Figure S5. Calibration curves for smartphone analysis of lateral flow immunoassays using background subtraction. Smartphone (Huawei P20) analysis for lateral flow immunoassay Peanut Hazelnut Control (PHC) and Hazelnut Peanut Control (HPC) assays, plotted as a function of the concentration of Total Hazelnut Protein (THP) and Total Peanut Protein (TPP) spiked into Running Buffer (RB) (100–0.05 ppm). Where LAB values are obtained by subtraction of the background nitrocellulose values from the test lines. (**A**) PHC in 1 µL sample: 99 µL Running Buffer (RB) (**B**) PHC in 25 µL sample: 75 µL RB (**C**) PHC in 75 µL sample: 25 µL RB (**D**) HPC in 1 µL sample and 99 µL RB (**E**) HPC in 25 µL sample: 75 µL RB. Error bars represent the standard deviation (n = 3). For experimental details, see the full manuscript.

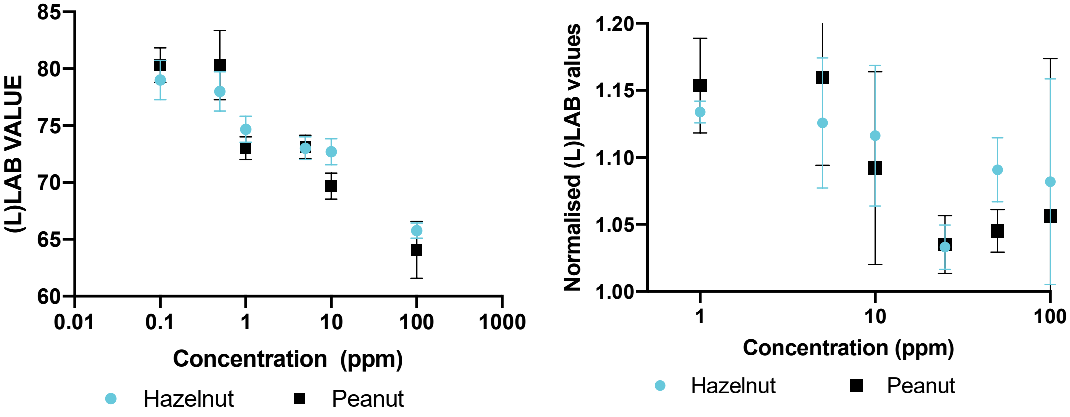

(**A**) (**B**)

Figure S6. Calibration curves for smartphone analysis of active flow-through immunoassay. Smartphone (Huawei P20) analysis of active flow-through immunoassay in a decreasing concentration of Total Hazelnut Protein (THP) and Total Peanut Protein (TPP) spiked in Running Buffer (RB) (100–0.01 ppm) all flow-through assays performed and analyzed in triplicate. (**A**) Analysis performed by subtracting the background reading from test spots. (**B**) Analysis performed by normalizing L (LAB) values (test line intensity/control line intensity; T/C). Error bars represent standard deviation (n = 3) For experimental details, see the full manuscript.

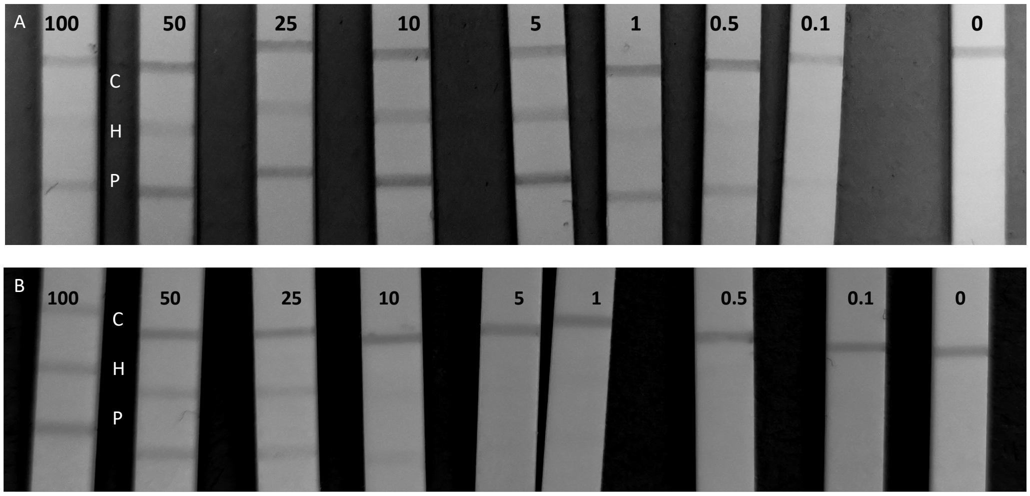

Figure S7. Optimized lateral flow immunoassay calibration curve in spiked matrix extract. Peanut Hazelnut Control (PHC) format Lateral Flow Immunoassay tested in a decreasing concentration (100–0.1 ppm) of Total Hazelnut Protein (THP) and Total Peanut Protein (TPP) spiked in matrix extract. The control region is indicated by C, the hazelnut detection region by H and the peanut detection region by P. (**A**) PHC using optimized assay under optimized conditions of 25 μL biscuit matrix extract spiked with THP, and TPP and 75 μL Running Buffer (RB). (**B**) PHC using optimized assay using 1 μL of biscuit matrix spiked with THP, TPP and 99 μL of RB conditions. For experimental details, see the full manuscript.

Table S1. Ingredient and allergen information for the 20 varieties of biscuit used for matrix extract experiments.

Sample Number	Ingredients	Allergen Information
1	Sugar, palm oil, glucose–fructose syrup, salt, raising agents: sodium carbonates, ammonium carbonates	40% oat flakes, wholegrain wheat flour, wheat flour<br><br>**May contain:** milk, sesame
2	Sugar, sunflower oil, glucose–fructose syrup, raising agent (citric acid [E330], sodium carbonate [E500], ammonium carbonate [E503]), salt	33% whole wheat flour, 19% wheat flour, 11% wheat flake<br><br>**Contains**: gluten<br>**May contain**: milk, sesame
3	Sugar, 15% palm oil, glucose–fructose syrup, salt, raising agent (sodium carbonate [E500]), ammonium carbonate [E503])	46% wheat flour, wholemeal wheat flour, oat flake<br><br>**Contains:** port gluten, wheat gluten<br><br>**May contain:** milk, sesame
4	Sugar, vegetable oil (palm, turnip), glucose syrup, leavening agent (diphosphate [E450], sodium carbonate [E503], mono and diglycerides of fatty acids, esterified with	Oat flake, wheat flour, wholemeal wheat flour, barley malt flour<br><br>**Contains:** port gluten, wheat gluten, gluten

	monoacetyl and diacetyl tartaric acid [E472e molasses, salt cane, salt	**May contain:** egg, gluten contain cereals, milk, nuts, peanuts
5	Maize starch, palm fat, cane sugar (16%), maize flour, buckwheat flour (4%), sugar beet syrup, modified tapioca starch, salt, raising agents (ammonium hydrogen carbonate, sodium hydrogen carbonate)	Gluten and Lactose free<br><br>Soya flour, soya bran (7%)<br><br>**Contains:** soy<br>**May contain:** lupin
6	Sugar, palm oil, salt, glucose–fructose syrup, raising agent, (sodium carbonate [E500], ammonium carbonate [E503]), natural flavor	Wheat flour<br><br>**Contains:** wheat gluten<br>**May contain:** milk, sesame
7	Corn starch, vanilla (sugar, corn starch, vanilla extract), sea salt, raising agent: ammonium carbonates	Wheat flour, butter (26%), free-range eggs<br><br>**Contains:** wheat gluten, milk, eggs<br><br>**May contain:** soy, almonds, cashews, hazelnut
8	Wheat flour, Sugar, palm oil, salt, glucose–fructose syrup, raising agent, (ammonium carbonate [E503]), natural flavor	**Contains:** wheat gluten<br><br>**May contain:** milk, sesame
9	Maize starch, butter (milk), palm fat, sugar, maize flour, maize starch, sugar beet syrup, modified tapioca starch, salt, whole milk powder, emulsifier (mono- and diacetyl tartaric acid) raising agents (ammonium hydrogen carbonate, sodium hydrogen carbonate)	Gluten free<br><br>**Contains:** milk, eggs<br><br>**May contain:** soya, lupin
10	Maize starch, maize flour, vegetable margarine [vegetable fats and oils (palm, palm kernel, rape seed)] water, salt emulsifier: mono- and diglycerides of fatty acids (E471); natural flavoring, maltodextrin, modified tapioca starch, sea salt, 2% rice syrup, raising agents: ammonium hydrogen carbonate (E503ii), sodium bicarbonate (E500ii); glucose. Syrup, yeast emulsifier, citric acid, maize starch	Gluten free<br><br>Soya protein, soya flour<br><br>**Contains:** soya<br><br>**May contain:** lupin
11	Sugar, palm oil, glucose–fructose syrup, salt, raising agents: sodium carbonates, ammonium carbonates	65% oat flakes, barley malt extract<br><br>**Contains:** gluten<br><br>**May contain:** sesame seeds, milk
12	Sugar, palm oil, glucose–fructose syrup, salt, raising agents: sodium carbonates, ammonium carbonates, emulsifier lecithin, antioxidant: sodium disulfite	65.5% wheat flour, lactose (milk), lecithin (soya)<br><br>**Contains:** wheat gluten, milk, soy<br><br>**May contain:** sesame seed
13	Sugar, vegetable oil (palm), glucose syrup, raising agents (sodium carbonates, disodium	Wheat flour, lactose and milk proteins

	diphosphate, ammonium carbonates), salt, aromas	**Contains:** gluten, milk<br><br>**May contain:** egg, soya, sesame seed
14	Sugar, palm oil, inverse sugar syrup, salt, raising agent, (sodium carbonate [E500], ammonium [E503])	40% oat flakes, 18% wholemeal wheat flour<br><br>**Contains:** oat gluten, wheat gluten<br><br>**May contain:** milk, sesame
15	Gluten free oat flakes 42%, sugar, gluten free oatmeal 17%, vegetable oil (palm), partially inverted sugar syrup, raising agent (sodium carbonates), salt	*Gluten free*<br><br>**May contain:** milk, egg, soya
16	Palm oil, sugar, inversion syrup, salt, raising agents: sodium carbonates, citric acid, ammonium carbonates	48% wheat flour, 14% whole wheat flour<br><br>**Contains:** wheat gluten<br><br>**May contain:** sesame seed, milk
17	Sugar, palm oil, glucose syrup, raising agents (sodium carbonates, disodium diphosphate, ammonium carbonates), salt, aromas	Wheat flour, milk powder<br><br>**Contains:** wheat gluten, milk<br><br>**May contain:** egg, soya
18	Sugar, 10% palm oil, glucose–fructose syrup, salt, raising agent (sodium carbonate, ammonium carbonate)	40% wheat flour, wholemeal wheat flour, oat flake<br><br>**Contains:** gluten, wheat gluten<br><br>**May contain:** milk, sesame seeds
19	Sugar, palm oil, salt, glucose–fructose syrup, raising agents: sodium carbonates, ammonium carbonates	60% oat flakes, barley malt extract<br><br>**Contains:** gluten<br><br>**May contain:** sesame seeds, milk
20	Sugar, palm oil, salt, glucose–fructose syrup, raising agent, (ammonium carbonate, sodium carbonates) natural aromas	Wheat flour, oat flakes<br><br>**Contains:** wheat gluten<br><br>**May contain:** milk, sesame

*See manuscript for full details.
